# Application of Chemometrics Tools to the Study of the Fe(III)–Tannic Acid Interaction

**DOI:** 10.3389/fchem.2020.614171

**Published:** 2020-12-11

**Authors:** Silvia Berto, Eugenio Alladio

**Affiliations:** Department of Chemistry, University of Turin, Turin, Italy

**Keywords:** tannic acid, iron(III), chemometric techniques, spectrophotometry, fluorescence, coordination compounds

## Abstract

Chemometric techniques were applied to the study of the interaction of iron(III) and tannic acid (TA). Modeling the interaction of Fe(III)–TA is a challenge, as can be the modeling of the metal complexation upon natural macromolecules without a well-defined molecular structure. The chemical formula for commercial TA is often given as C_76_H_52_O_46_, but in fact, it is a mixture of polygalloyl glucoses or polygalloyl quinic acid esters with the number of galloyl moieties per molecule ranging from 2 up to 12. Therefore, the data treatment cannot be based on just the stoichiometric approach. In this work, the redox behavior and the coordination capability of the TA toward Fe(III) were studied by UV-vis spectrophotometry and fluorescence spectroscopy. Multivariate Curve Resolution-Alternating Least Squares (MCR-ALS) and Parallel Factor Analysis (PARAFAC) were used for the data treatment, respectively. The pH range in which there is the redox stability of the system Fe(III)–TA was evaluated. The binding capability of TA toward Fe(III), the spectral features of coordination compounds, and the concentration profiles of the species in solution as a function of pH were defined. Moreover, the stability of the interaction between TA and Fe(III) was interpreted through the chemical models usually employed to depict the interaction of metal cations with humic substances and quantified using the concentration profiles estimated by MCR-ALS.

## Introduction

Tannic acid (TA) is a naturally derived polyphenolic compound. It belongs to the class of hydrolyzable tannins (Barbehenn and Peter Constabel, 2011; Rhodes, 2020) that are natural polymers derived from the vegetable kingdom. TA possesses diverse bonding abilities. It is able to complex or cross-link macromolecules through multiple interactions, such as hydrogen and ionic bonding and hydrophobic interactions (Heijmen et al., 1997; Shutava et al., 2005; Erel-Unal and Sukhishvili, 2008). It can also coordinate metal ions through the oxygenated functions and the coordination capability was exploited to form TA–metal networks (Ejima et al., 2013; Guo et al., 2014). The molecule of TA is based on a α/β-D-glucopyranose skeleton whose hydroxyl groups are partially esterified by gallic acid (GA, 3,4,5-trihydroxybenzoic acid). The GA molecules form chains composed of two or more units linked together by ester bonds. The chemical formula for commercial TA is often given as C_76_H_52_O_46_, which corresponds to decagalloyl glucose, with a 1,701.20 g mol^−1^ molecular weight, but in fact, it is a mixture of polygalloyl glucoses or polygalloyl quinic acid esters with the number of galloyl moieties per molecule ranging from 2 up to 12 depending on the plant source used to extract the TA (Arapitsas et al., 2007). Despite this, TA is used successfully in many application fields without further purification or separation of the single components (Albu et al., 2009; Gülçin et al., 2010; Ejima et al., 2013), suggesting that the main chemical properties of the commercial products are quite similar.

The TA shows affinity toward iron cations since its numerous oxygenated functions. A work of J.D. Hem, published in the 1960, titled “Complexes of ferrous iron with tannic acid” (States and Printing, 1960) attests that this capability is known for a long time. Notwithstanding, the interpretation of the interaction between the Fe(III) and TA in aqueous solution still presents many deficiency. There are ambiguous information about the redox behavior of Fe(III) in the presence of TA as a function of pH. It is known that TA shows reduction capability toward the Fe(III) cation (States and Printing, 1960), as it is common for polyphenolic molecules (Kipton et al., 1982), and its reduction capability is higher in acidic conditions (States and Printing, 1960). Nevertheless, some recent works dealing about the complexation of iron cations by TA reported the formation of coordination compounds of Fe(III) and TA at very low pH (Sungur and Uzar, 2008; Fu and Chen, 2019).

Modeling the interaction of Fe(III)–TA is a challenge, as can be the modeling of the metal complexation upon natural macromolecules, such as Humic Substances (HS) (Filella and Hummel, 2011). Unlike HS, however, TA shows a restricted variety of binding sites. TA can interact with metal cations through the phenolic groups, which only differ each other for the position in the polymer structure. Moreover, it is possible to assign to TA a molecular weight mean value, which allows calculating a theoretical value of the molar concentration and, therefore, using experimental protocols usually suitable for low-molecular-mass ligands. A previous study was conducted on the protogenic properties of TA (Ghigo et al., 2018) revealing that its protogenic behavior can be explained by a model that supposes the presence of gallic acid and of three different types of phenolic functions. The protonation constants estimated correspond to the dissociation of different types of phenolic groups but cannot be attributed to a protogenic function with a univocal position in the ligand molecule (Ghigo et al., 2018) as well as for low-molecular-mass ligands. The work was carried out combining potentiometry, UV-vis and fluorescence spectroscopy, as well as *ab initio* calculations. The experimental data were treated by both thermodynamic and chemometric approaches, and the Multivariate Curve Resolution-Alternating Least Squares (MCR-ALS) technique turned out to be useful in the interpretation of the spectroscopic data and, in particular, in differentiating the contribution of GA on the fluorescence spectra of TA.

In this work the redox behavior and the coordination capability of the TA toward Fe(III) were studied by UV-vis spectrophotometry and fluorescence spectroscopy. Unfortunately, the use of potentiometry for the study of the Fe(III)–TA system is not allowed because of the low solubility of the coordination compounds formed. The concentration permitted are in the order of 10^−5^ mol L^−1^, values not proper for the potentiometric technique sensitivity.

Since TA is actually a mixture of different polymeric structures, the most conventional hard-modeling methods, based on molar concentration and mass/charge balance equations, may not be the best way to define the type and the strength of the interaction between TA and Fe(III); therefore, chemometric ways (soft-modeling methods) were tested. Since MCR-ALS technique turned out useful for the interpretation of TA protonation (Ghigo et al., 2018), it was used also here for the spectroscopic data treatment, together with Parallel Factor Analysis (PARAFAC). MCR-ALS and PARAFAC are probably the most used multivariate analysis methods to study chemical solution equilibria (de Juan and Tauler, 2003; Jaumot et al., 2011; Ruckebusch and Blanchet, 2013). These methods obtain to recover information without any assumption about the stoichiometry of the species involved or law that governs the chemical reaction. The aim of the work was to evaluate (*i*) the pH range in which there is the redox stability of the system Fe(III)–TA, (*ii*) the binding capability of TA toward Fe(III), (*iii*) the spectral features on coordination compounds, and (*iv*) the concentration profiles of the species in solution as a function of pH. The stability of the interaction between phenolic moieties of the TA and Fe(III) was interpreted through the chemical models usually employed to depict the interaction of metal cations with humic substances.

## Materials and Methods

### Chemicals

TA (puriss.), 1-10-phenanthroline (≥99%), iron(III) nitrate nonahydrate (ACS reagent, ≥98%), potassium nitrate (≥99.0%), nitric acid (65%), and sulfuric acid (95–97%) were from Sigma-Aldrich (St. Louis, Missouri, USA). A stock solution of Fe(NO_3_)_3_ 1 × 10^−3^ mol L^−1^ was prepared dissolving iron(III) nitrate nonahydrate in HNO_3_ 0.01 mol L^−1^ in order to avoid the precipitation of metal hydrolytic species. A stock solution of TA 1 × 10^−3^ mol L^−1^ was prepared daily in ultrapure water, to avoid the degradation of the organic compound. Potassium hydroxide and hydrochloric acid solutions used as titrant, or for adjusting pH, were prepared by diluting Merck (Darmstadt, Germany) concentrated products. The concentration of the potassium hydroxide solution was assessed by standardization against potassium hydrogen phthalate (Sigma-Aldrich). The purity and the title of the used acids were evaluated by pH-metric titrations. Ultrapure water (Milli-Q, Millipore) was used to prepare all the solutions.

### Apparatuses

A Metrohm (Herisau, Switzerland) potentiometer (model 713, resolution of ± 0.1 mV) was used for pH measurements. It was coupled with Metrohm 765 Dosimat burettes (minimum deliverable volume of ± 0.001 cm^3^) and equipped with Metrohm combined glass electrodes (mod. 6.0259.100). The temperature control (25.0 ± 0.1°C) was achieved by means of water circulation, in the outer chamber of the titration cell, from a thermocryostat (mod. D1-G Haake, Victoria, Australia).

The absorption spectra were recorded with a Jasco (Cremella, LC, Italy) double-beam spectrophotometer UV-vis, model V-550, equipped with Hellma (Jena, Germany) quartz cuvettes (1.000 cm optical path length). In order to record the spectra as a function of pH, the solutions under alkalimetric titration were transferred to and from the optical cell by a peristaltic pump (model SP311, VELP Scientifica, Usmate, MB, Italy).

A Varian (Segrate MI, Italy) Cary Eclipse fluorescence spectrofluorometer was used to record the fluorescence excitation–emission matrix (EEM) spectra with Hellma cuvettes (1.000 × 1.000 cm optical path lengths).

### Procedures

The electrode couple was daily calibrated in terms of H^+^ concentration by titrating a 5 × 10^−3^ mol L^−1^ HCl solution at the working ionic strength (0.001 mol L^−1^, KNO_3_) with standard KOH. In this way, we can assess the slope and the formal potential *E*^0^ of the Nernst equation in conditions like those of the sample solutions under study. In order to avoid O_2_ and CO_2_ contamination during the titration, a stream of purified N_2_ was bubbled in the titration cell.

The spectrophotometric titrations were carried out on 50 ml of solutions of Fe(III)–TA, with cation concentrations included between 2.0 × 10^−5^ and 8.0 × 10^−5^ mol L^−1^ and metal-to-ligand ratios comprised between 1:1 and 3:1. The titrant was 0.1 mol L^−1^ KOH standard solutions. The TA concentration was calculated based on the formal molecular weight. The ionic strength was 0.001 mol L^−1^, and it was obtained considering the different ionic components of the solution and the eventual addition of potassium nitrate to reach the exact value. The addition of higher amount of salt would have been preferable in order to maintain the ionic strength at a fixed value during the titration process, but it was not possible because it leads to the formation of a solid purple compound, probably for salting out effect. The titrations were conducted from pH ~3.5 to pH 8.5 because, as explained below, this is the pH range in which it is possible to exclude the presence of redox equilibria. The spectra were recorded in the wavelength range 400–900 nm and a baseline was taken in air before each absorbance measurement. Each absorbance spectrum was taken against the reference cuvette filled with Milli-Q water. UV-vis spectra were also recorded on solutions with different metal-to-ligand ratios, at fixed pH, in order to estimate the metal binding capacity of TA.

The fluorescence EEMs were taken with excitation wavelengths in the range of 200–500 nm, at 10 nm intervals, and emission wavelengths from 250 to 600 nm. A 10 nm bandpass was adopted on both excitation and emission. Solutions with Fe(III) 5.0 × 10^−6^ mol L^−1^ and TA 5.0 × 10^−6^ mol L^−1^, at pH 4.0, 4.2, 4.4, 5.2, 5.7, 5.9, 6.2, 6.7, 7.1, and 8.1, or with only TA 5.0 × 10^−6^ mol L^−1^, at pH 4.0, 5.0, 6.0, 7.0, and 8.0, were analyzed. The pH of the solutions was adjusted with KOH. The Raman signal of water was taken as a reference for signal stability within different measurements.

### Data Handling

#### Calibration Data Analysis

The electrode calibration data were elaborated by the ESAB2M program (De Stefano et al., 1987) in order to refine the electrode parameters: formal potential *E*^0^, Nernstian slope, and analytical concentration of reagents.

#### Multivariate Curve Resolution–Alternating Least Squares Regression (MCR-ALS)

The UV-vis spectra were evaluated by Multivariate Curve Resolution–Alternating Least Squares regression (MCR-ALS) chemometric approach, whose goal is to decompose the collected data into their pure chemical components, by providing their spectra and their concentration profiles (Tauler, 1995; de Juan et al., 2000; Jaumot et al., 2005, 2015; Ruckebusch and Blanchet, 2013). The MCR-ALS approach has been deeply used in several applications such as, for instance, voltammetry (Serrano et al., 2020), UV-vis (Veselinović et al., 2012; Ghigo et al., 2018), IR (Shariati-Rad and Hasani, 2009), Raman (Andrew and Hancewicz, 1998; Lyndgaard et al., 2013), hyperspectral imaging (Piqueras et al., 2011; Laborde et al., 2021), NMR (Huo et al., 2004), EPR (Abou Fadel et al., 2014; Berto et al., 2019), GC-MS (Lebanov et al., 2020), UHPLC (Wehrens, 2011), etc. It is particularly helpful in case of measurements following Lambert-Beer's law, i.e., in case the collected overall spectra consist in a linear combination of the spectra of their pure components. Briefly, MCR-ALS deconvolution is made as follows (Equation 1):

(1) $$\begin{array}{l} X=CS^{T}+E \end{array}$$

where *X* represent the collected data matrix, *C* is the matrix of the pure concentration profiles, *S*^*T*^ is the transposed matrix of the pure spectra, and *E* is the residuals (error) matrix. Therefore, each *x* collected spectrum constituting the *X* matrix can be written, as follows (Equation 2):

(2) $$\begin{array}{l} x=c_{1}s_{1}^{T}+...+c_{n}s_{n}^{T} \end{array}$$

where *c*_1_, ⋯ , *c*_*n*_ represent the concentrations of the *n* pure components/chemical species for the evaluated *x* mixture, while *s*_1_, ⋯ , *s*_*n*_ stand for the spectra of the *n* pure components. Consequently, this approach can be easily extended to all the collected spectra of the samples included into the *X* matrix. The first step of MCR-ALS decomposition is represented by the initial estimation of the set of pure concentrations *C* or the pure spectra *S*. This initial estimation can be performed by means of several algorithms such as, for instance, Evolving Factor Analysis (EFA) (Maeder, 1987), Evolving Windowed Factor Analysis (EWFA) (Keller and Massart, 1991), Orthogonal Projection Approach (OPA) (Sánchez et al., 1994), and SIMPLISMA (Windig et al., 2012). It has to be noted that it is useful to spend some time in obtaining satisfactory initial estimates since, if they show a good quality in terms of results, ALS algorithm (that consists in a repeated application of multiple least squares regression) will need a quite small number of iterations before reaching a convergence. In fact, once an initial estimate of, for instance, *S* has been obtained, the initial *C* matrix is calculated by ALS by minimizing the residuals *E*. The described process is performed several times until an optimal convergence value is obtained. Furthermore, several constraints can be adopted in the MCR-ALS calculation in accordance with the chemical and physical properties of the samples and the data under investigation (Wehrens, 2011). They are functions that can be applied separately to the estimates of the spectra or the concentration profiles on every step of the algorithm, aiming to minimize the overall error. In the case of UV-vis spectra, for instance, a non-negativity constraint can be applied to both the pure concentration profiles (i.e., no negative concentrations) and the pure spectra (no negative signals). Further constraints can be implemented such as, for instance, (*i*) a closure constraint, which is usually adopted for the contribution profiles and aims at conserving the mass balance (i.e., the overall sum of the concentrations of the chemical compounds in the mixture is constant); (*ii*) a unimodality constraint, which is employed to let the reconstituted signals having only one maximum; (*iii*) a normalization constraint, whose goal is to normalize the pure spectra or the concentration profiles of the *n* components to a specific reference value; (*iv*) an angle constraint, which can be set in order to enhance the contrast among the obtained solutions (Windig et al., 2012).

#### Parallel Factor Analysis (PARAFAC)

The fluorescence EEMs were elaborated by Parallel Factor Analysis (PARAFAC, also known as canonical decomposition, CANDECOMP) chemometric approach (Carroll and Chang, 1970; Smilde, 1992; Bro, 1997). PARAFAC is undoubtedly one of the most frequently used approach for deconvolving EEM (Leurgans and Ross, 1992; Murphy et al., 2013). PARAFAC's main goal is the identification of the independent species (named components) that are present into the collected data (in this case, EEM). This is achieved by performing a decomposition of the trilinear multi-way data arrays, but the PARAFAC approach can be adopted also with higher-order arrays [since it belongs to the group of multivariate modeling known as multi-way methods (Murphy et al., 2013)]. The PARAFAC approach is particularly helpful when evaluating EEM data since the independent components that are obtained during the decomposition can be chemically interpreted. In fact, EEM data follow Lambert–Beer's law since the measured fluorescence shows a quite linear correlation with the concentrations of the *N* chemical compounds constituting the analyzed samples. Consequently, EEM data can be considered as trilinear since emission and excitation spectra are independent of one another, and the recorded fluorescence can be seen as a sum of the signals of the different *N* components.

The overall PARAFAC trilinear decomposition is, as follows (Equation 3):

(3) $$\begin{array}{l} x_{ijk}=\overset{N}{\underset{n=1}{\sum }}a_{in}b_{jn}c_{kn}+e_{ijk} \end{array}$$

where *a, b*, and *c* represent the three terms that are provided by the decomposition, while *e*_*ijk*_ represents the residual array (i.e., the difference between the original three-way dataset and the one that has been obtained after the PARAFAC decomposition). *i, j*, and *k* stand for the three modes of a three-way dataset so that, for EEM data, the element *x*_*ijk*_ represents the *i*^th^ sample (mode 1) for the *j*^th^ emission variable (mode 2) and the *k*^th^ excitation variable (mode 3). Finally, *n* is the number of independent species/components that can extracted from the original data during the deconvolution process. Therefore, each *n* component has its own *i, j*, and *k* values for each sample, emission wavelength, and excitation wavelength. The analyst must define the proper number of *n* components composing the EEM data by evaluating when they significantly deviate from Lambert–Beer's law (Bro, 1997; Murphy et al., 2013).

## Results

### Redox Behavior

The instability of TA in alkaline conditions was previously tested (Ghigo et al., 2018). The UV-vis spectra at alkaline pH showed the instability of TA for pH higher than 8.5.

**Figure 1:**
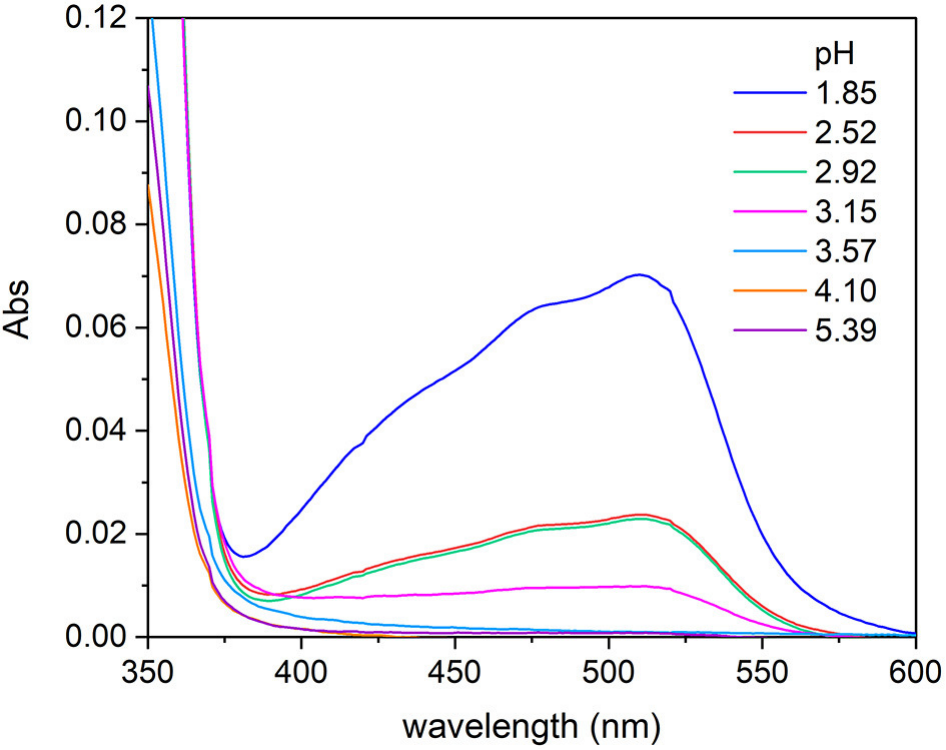
Absorption spectra recorded on solutions of Fe(III)/1,10-phenantroline/TA with concentrations 2.0 × 10^−5^, 6.5 × 10^−5^, and 4.0 × 10^−5^ mol L^−1^, respectively, at different pH, after 2 h from the preparation.

The TA also shows reducing capability, particularly in acidic conditions, and this could lead to the reduction of Fe(III) to Fe(II) present in solution as reported by Kipton et al. (1982) for GA and its homologs. In order to assess this redox behavior and to identify the pH threshold below which the phenomenon can happen, some experiments were carried out. A series of solutions of Fe(III) 2.0 × 10^−5^ mol L^−1^, 1,10-phenantroline 6.5 × 10^−5^ mol L^−1^, and TA 4.0 × 10^−5^ mol L^−1^ were prepared. The pH was controlled adding H_2_SO_4_ 1.0 mol L^−1^ or KOH 1.0 mol L^−1^, in order to prepare solutions with pH comprised between 1.8 and 8.5. The UV-vis spectra of the solutions were recorded after 2 h from the preparation, the time needed for a stable color development. For the solutions in which the Fe(III) was reduced to Fe(II), the red color appeared because of the formation of the complex Fe(II)-1,10-phenantroline that shows an absorption maximum at about 510 nm. The spectra collected highlighted that the reduction of Fe(III) to Fe(II) induced by TA starts for pH lower than 3.5. It is possible to observe in Figure 1 that for pH 3.57, the signal typical of the complex Fe(II)-1,10-phenantroline is absent, whereas for pH 3.15, it is clearly detectable, although the solutions are kept in the presence of oxygen. For this reason, the investigation of the coordination capability of TA toward Fe(III) was conducted in the pH range 3.5–8.5. This result agrees with the statements reported by Kipton et al. (1982).

### UV-Vis Spectra

**Figure 2:**
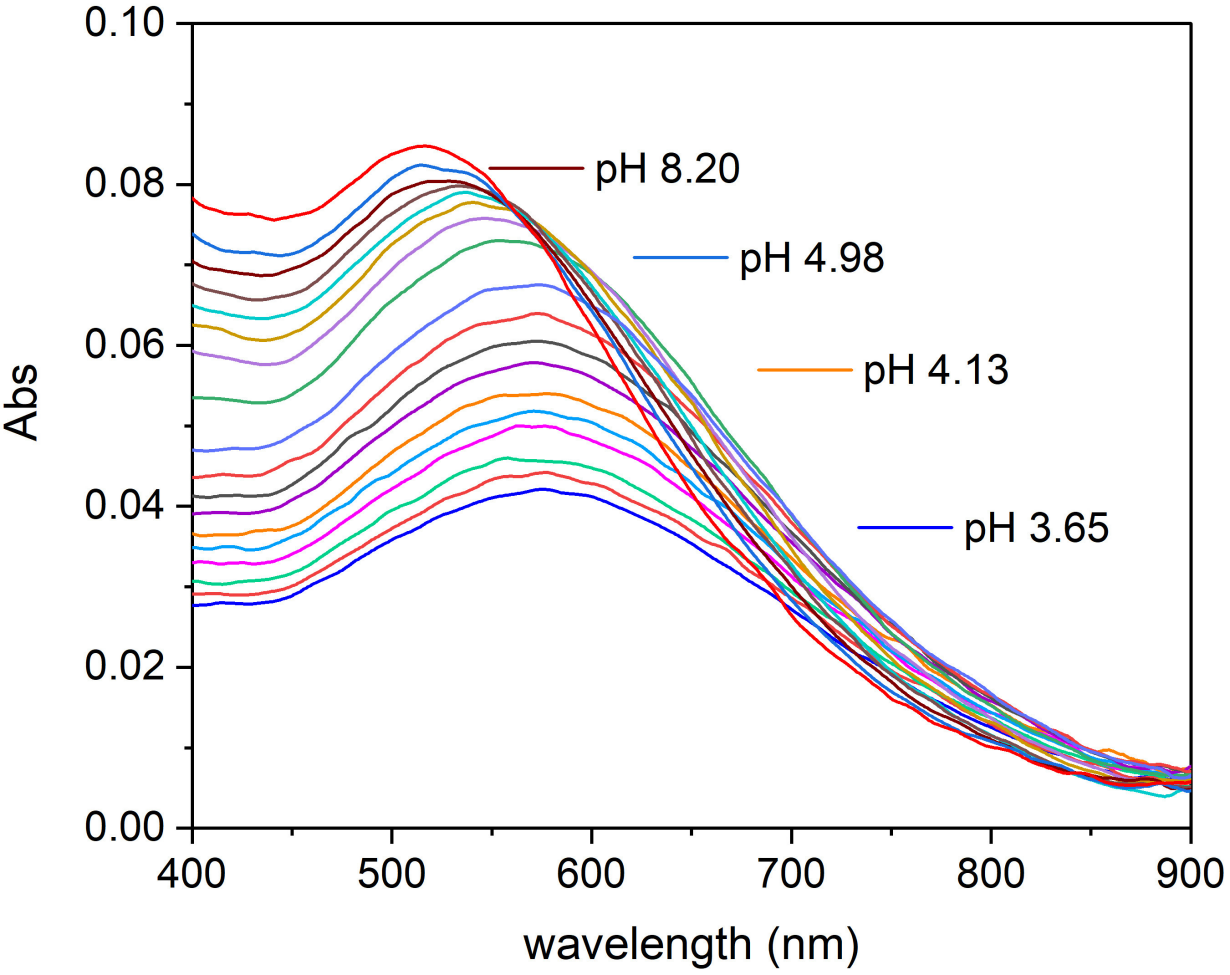
Absorption spectra recorded on a solution of Fe(III)/TA with concentration 2.00 × 10^−5^ and 2.01 × 10^−5^ mol L^−1^, respectively, at different pH values.

Absorption spectra of Fe(III)–TA systems were recorded on solutions with different metal-to-ligand ratios. The spectra recorded on a solution with metal-to-ligand ratios 1:1 as a function of pH are shown in Figure 2. It is possible to note that the intensity of the absorption band with the maximum at 570 nm increases as the pH of the solution rises, until ~pH 5. After this value, there is a shift of the absorption band toward lower wavelengths and the color of the solution changes from dark purple to wine-red. This behavior is similar for all the Fe(III)–TA ratios studied.

The absorption maxima can be easily compared with those of catechol reported before. Sever and Wilker (Sever and Wilker, 2004) reported the wavelength maxima (λ_max_) for mono-, bis-, and tris-catecholate defined by themselves and by other authors. The Fe(III)–mono-catecholate complex shows two maxima at about 430 and 700 nm, but the bands are not well-defined, whereas the Fe(III)–bis-catecholate complex shows a better characterized absorption spectrum and, in particular, the scientists agree on the presence of a band at about 570 nm with an extinction coefficient ranged between 3,000 and 3,500 mol^−1^ L cm^−1^. Sever and Wilker (Sever and Wilker, 2004) observed that the deep blue–violet bis-catecholate was followed by a transformation to a wine-red colored species (isosbestic point at 547 nm), increasing the pH, and they supposed the formation of a tris-catecholate species. The Fe(III)–tris-catecholate complex has a band comprised between 470 and 490 nm with an extinction coefficient ranging between 3,700 and 4,320 mol^−1^ L cm^−1^ (Avdeef et al., 1978; Kipton et al., 1982; Sever and Wilker, 2004). This description is in accordance with what happens in the Fe(III)–TA system under study (Figure 2). The spectra of the species formed between pH 3.5 and 5 is coherent with a bis-catecholate coordination type. While the shift of the absorption maximum toward lower wavelengths with the pH increase is coherent with the Fe(III)–tris-catecholate spectral features, but it is not possible to exclude the deprotonation of a coordinated water molecule.

### Binding Capacity

**Figure 3:**
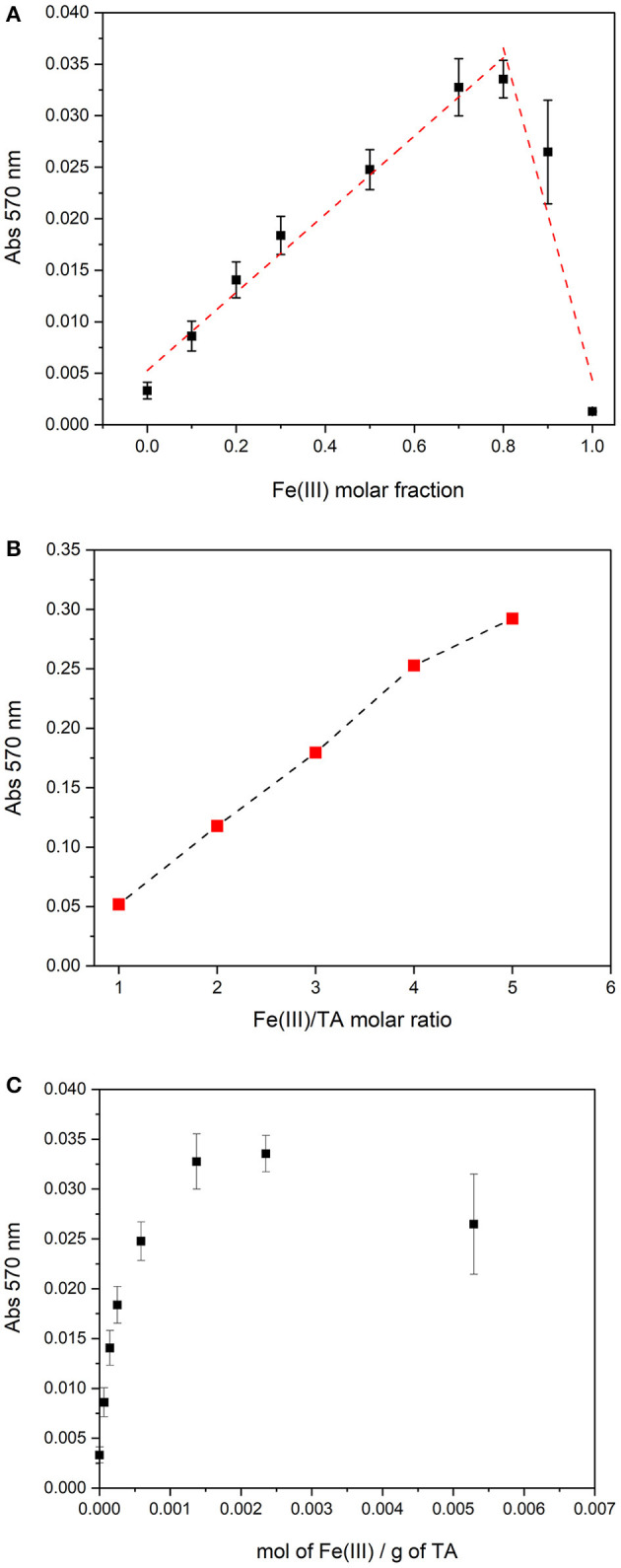
**(A)** Job's plot for solutions with a total concentration of 2.00 × 10^−5^ mol L^−1^ and pH = 4.0. **(B)** Absorbance at 570 nm of Fe(III)/TA solutions at pH ~4 with different molar ratios (TA concentration 2.00 × 10^−5^ mol L^−1^). **(C)** Absorbance at 570 nm of Fe(III)/TA solutions at pH = 4.0 as a function of the total mol of Fe(III) per total mass of TA.

Since the polymeric structure, TA can coordinate more than one Fe(III) cation. In order to evaluate the maximum number of iron cations that can be coordinated by a mole of TA, Job's plot method was used. Based on the spectral behavior, in the pH range comprised between 3.5 and 4.5, there is probably only a complex species; therefore, Job's method can be applied. Nine solutions of Fe(III) and TA with a total nominal concentration of 2.00 × 10^−5^ mol L^−1^, but with different molar fraction of the two components, were prepared, and the pH was adjusted at 4.0 with KOH. The absorption spectra of the solutions were recorded and the absorbance at 570 nm was plotted toward the molar fraction of Fe(III). The plot is reported in Figure 3A. The maximum of Job's plot is positioned at a molar fraction of 0.8, suggesting that TA can coordinate at most four Fe(III) cations. This result agrees with the observation that the Fe(III)–TA solutions with increasing metal-to-ligand ratio, from 1:1 to 5:1, at similar pH (pH ~4.0), showed absorbances that rise linearly with the metal-to-ligand ratio until a ratio of 4:1 (see Figure 3B). Therefore, the number of chromophores increases linearly with the concentration of Fe(III) until there are available binding sites. For molar ratios higher than 5, the precipitation occurs.

The data collected for Job's plot can be represented as absorbances *vs* mol Fe(III) per gram of TA in solution. As it is possibly observed in Figure 3C, the maximum absorbance value was reached for a ratio of 2.35 × 10^−3^ mol of Fe(III) per gram of TA. This notation allows the avoidance of the calculation of a molar concentration of TA, which may be considered as a forcing if we are working with a natural polymer such as TA.

### Fluorescence Spectra

**Figure 4:**
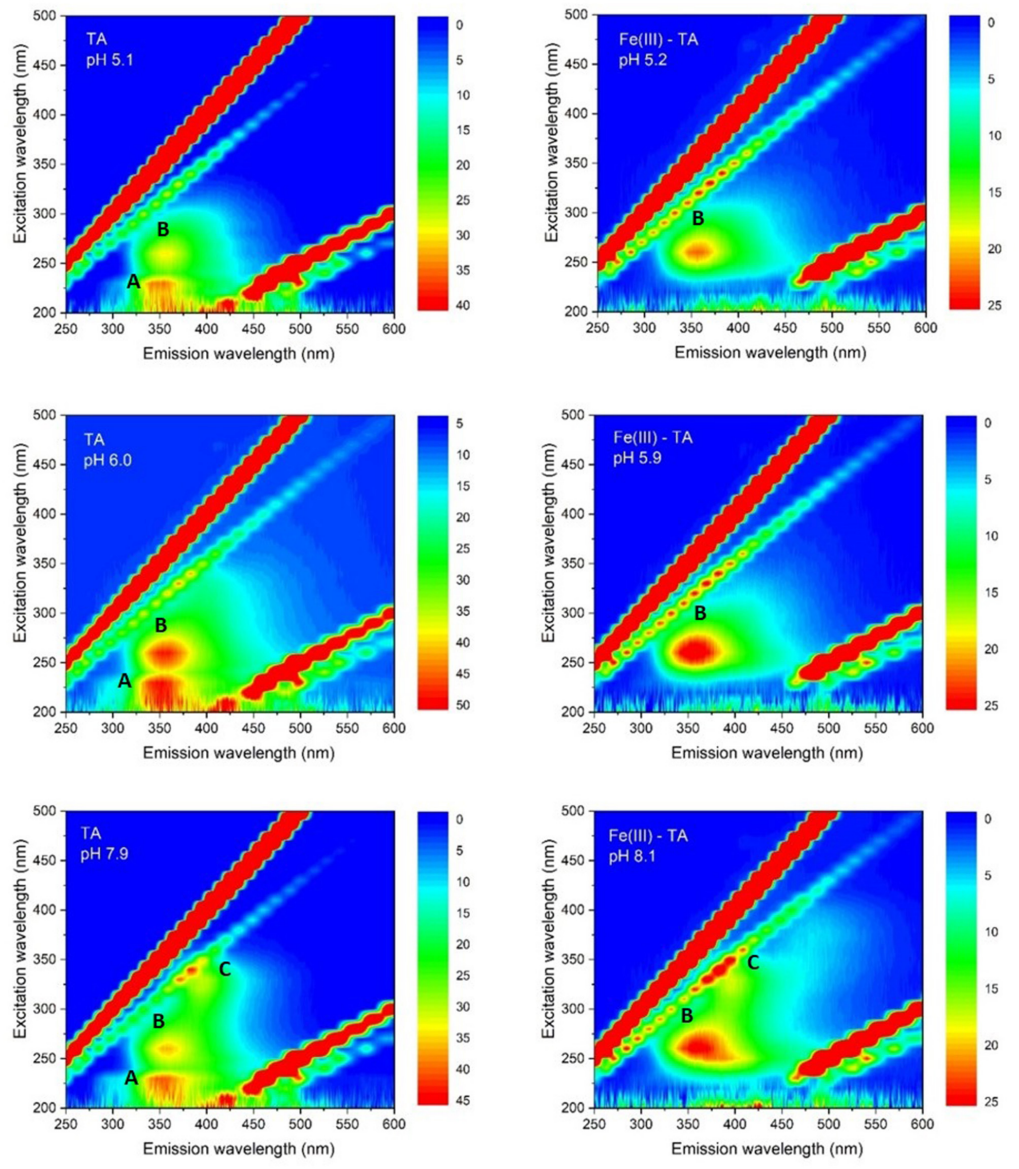
Contour plots of fluorescence EEMs recorded as a function of pH on water solutions of TA 5.0 × 10^−6^ mol L^−1^; Fe(III)/TA 5.0 × 10^−6^ /5.0 × 10^−6^ mol L^−1^, respectively.

**Figure 5:**
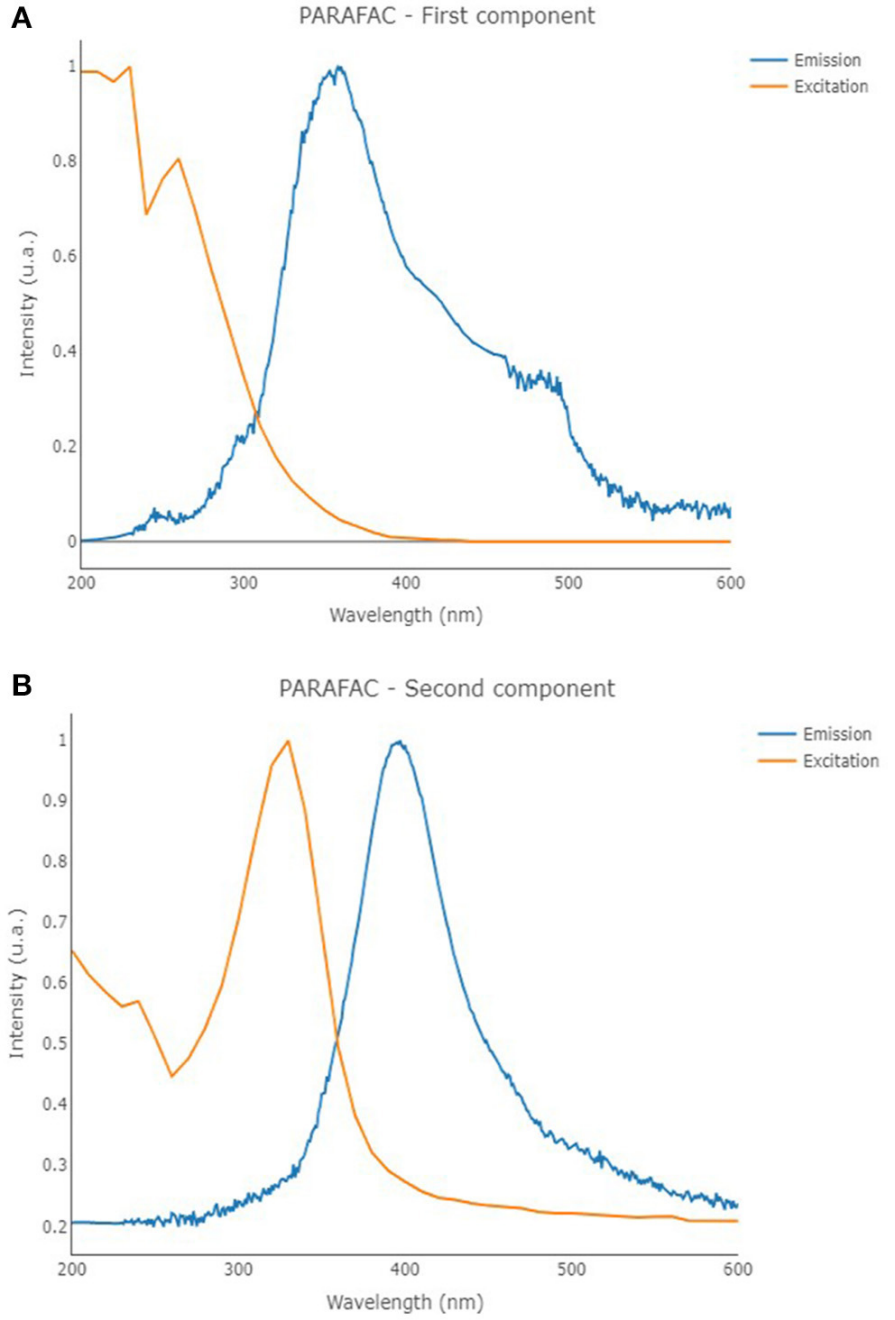
Excitation (orange line) and emission (blue line) profiles of the first **(A)** and second **(B)** components of the PARAFAC model that was built on the solutions containing only TA.

Fluorescence EEMs were taken on solutions containing TA and TA with Fe(III) at different pH. Figure 4 shows some fluorescence EEMs recorded on the two different systems. The fluorescence signals are quite different. The contour plots highlight that the emission peak of TA, located at excitation/emission wavelengths (Ex/Em) of 210/360 (i.e., peak A), is no longer visible in the presence of Fe(III). The solution containing the cation shows a defined emission peak at 260/358 nm (i.e., peak B), corresponding to the second emission peak of TA (Ghigo et al., 2018). The peak of TA at 325/395 nm (i.e., peak C), which is characteristic of pH higher than 8, is visible in both conditions, with and without the metal cation. A quenching effect of Fe(III) was previously detected on fluorescence signals of dissolved organic matter (DOM) (Poulin et al., 2014) and of soluble bio-organic substances (SBO) isolated from the organic fraction of solid urban wastes (Ballesteros et al., 2017). These results are (quite) confirmed by PARAFAC modeling. PARAFAC was performed by means of in-house code involving R software (version 4.0.2) (R Core Team, 2020) and the R packages *staRdom* (Pucher et al., 2019) and *eemR* (Massicotte, 2019). Two distinct PARAFAC models have been built, firstly on the EEM data involving the solutions containing only TA and then on those containing TA with Fe(III) (experimental details are reported in *Procedures* paragraph). EEM data were pre-processed. Different data pre-processing approaches were tested, before and after calculating the PARAFAC models, such as the removal of Raman and Rayleigh scattering and the Inner-Filter Effects (IFE—traditionally occurring when the chromophores absorb the excitation light). Only the removal of Raman and Rayleigh scattering provided good and interpretable results; therefore, only these results were reported. The removed scatter areas have also been interpolated [by means of splines (Lee et al., 1997)] to fasten the calculation. Both the PARAFAC models were built by calculating 50 starting models in order to achieve the global minimum, 5,000 maximum number of iteration steps, and a convergence value (tolerance) of 10^−6^. Non-negativity constraints were selected for all the modes, and both B and C modes were rescaled to a maximum fluorescence of 1 for all the evaluated components. Core consistency, *R*^2^, and number of iterations and residuals were evaluated as diagnostic parameters to define the proper number of components. The noisiness of the modeled components was considered, too (Bro, 1997; Murphy et al., 2013). Split-half analysis could not be performed since the number of evaluated samples was relatively low. The first PARAFAC model evaluating the solutions containing only TA provided a 2-component model (reported in Figure 5) showing an overall *R*^2^ of 0.921. The first component showed the excitation/emission peaks of TA, located at wavelengths of 210/360 nm. A shoulder peak is observed for the excitation spectrum around 260 nm, too. On the other hand, the second component showed the excitation/emission peaks that are characteristic of pH higher than 8, around 325/405 nm (actually, the original spectra of TA above pH = 8 showed an emission peak at 395 nm). Consequently, the PARAFAC model supported the results observed by evaluating the EEM data by indicating the existence of two different components with peculiar emission and excitation peaks.

**Figure 6:**
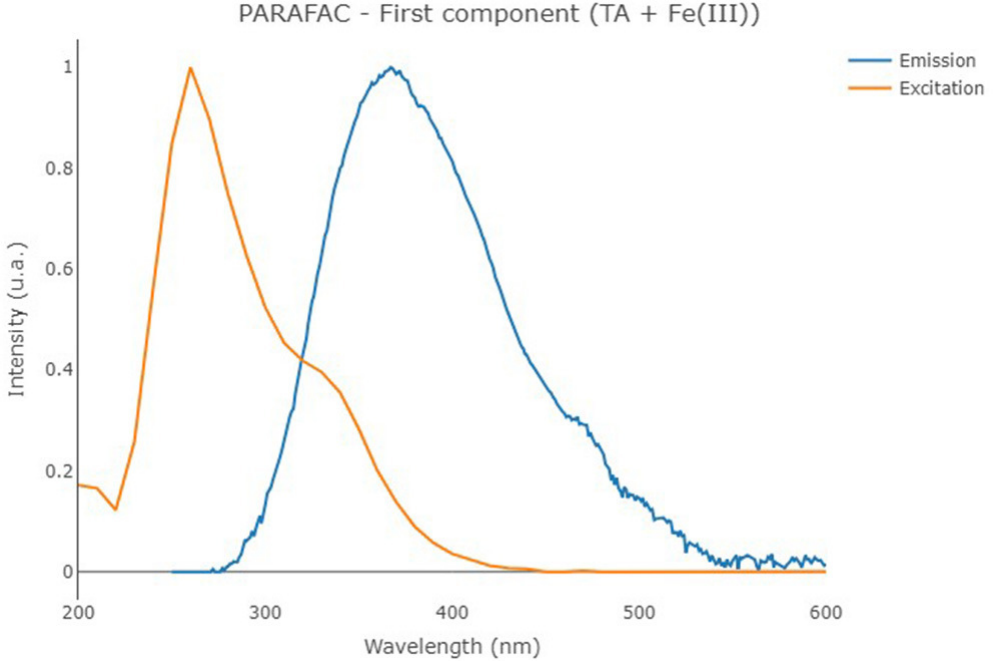
Excitation (orange line) and emission (blue line) profiles of the first component of the PARAFAC model that was built on the solutions containing TA with Fe(III).

The second PARAFAC model calculated on the solutions containing TA with Fe(III) provided a 1-component model (reported in Figure 6) showing an overall *R*^2^ of 0.833. In the present case, the solution containing the cation provided a component with an emission/excitation peak at 260/370 nm, roughly corresponding to the emission peak B of TA. In the present case, the peak C of TA at 325/395 nm, which is characteristic of pH higher than 8, is not noticed.

### MCR-ALS Results

**Figure 7:**
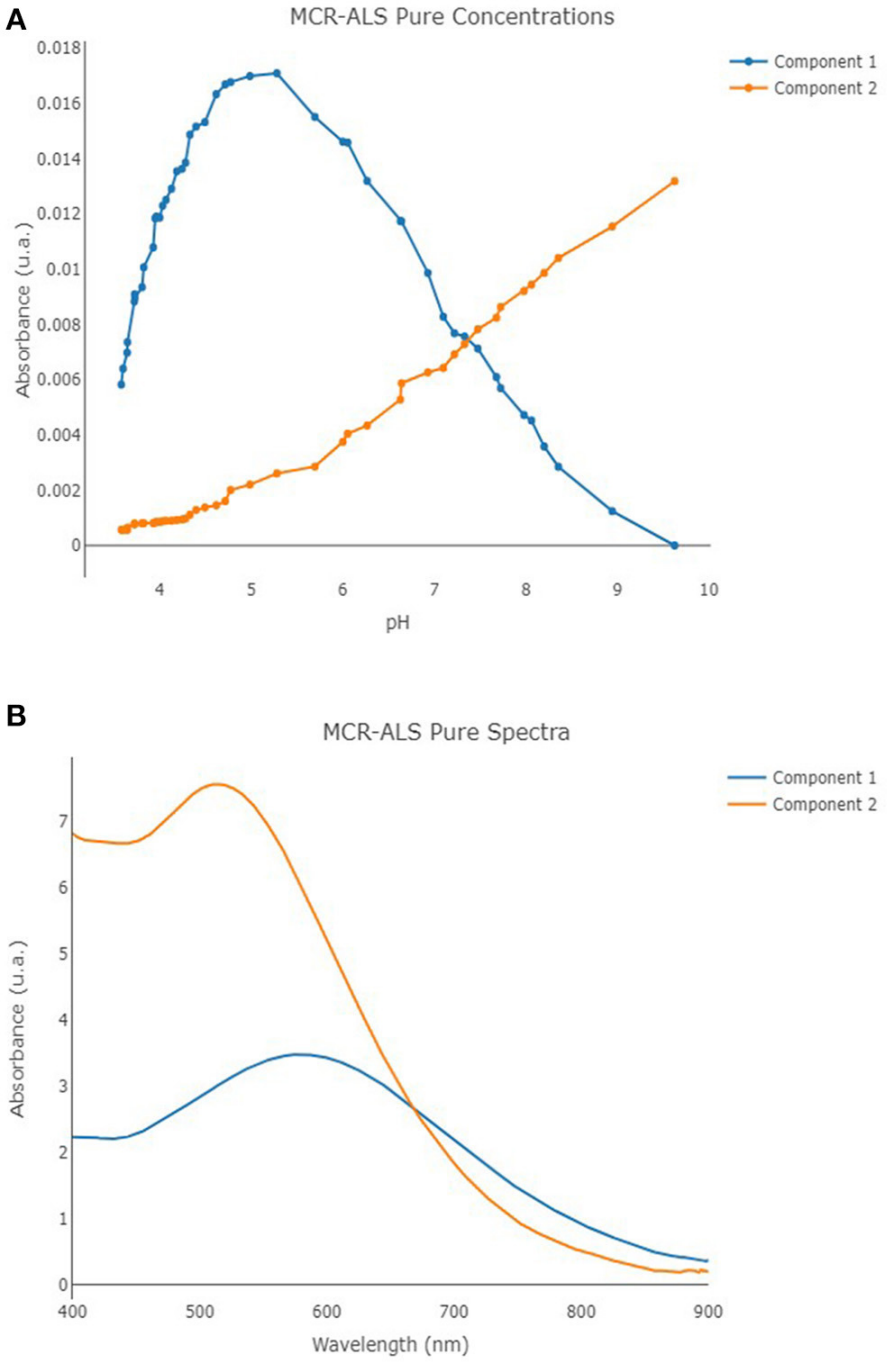
Component 1 (blue line) and component 2 (orange line) pure concentrations **(A)** and pure spectra **(B)** profiles for the developed MCR-ALS model.

MCR-ALS was performed once again by means of in-house code involving R software (version 4.0.2) (R Core Team, 2020) and the R package *mdatools* (Kucheryavskiy, 2020). The UV-vis spectra obtained on solutions with metal/ligand molar ratio 1:1 were analyzed by MCR-ALS technique. SIMPLISMA algorithm was employed to calculate the initial estimates, non-negativity constraints were selected for both the concentration profiles and the spectra, while a closure constraint was adopted for the concentration profiles by setting an overall concentration equal to 2.00 × 10^−^^5^ mol L^−1^. A total of 150 maximum number of iteration steps and a convergence value (tolerance) of 10^−6^ were defined, too. Two independent components were observed as the optimal number of components for the MCR-ALS model, involving an overall cumulative explained variance of 93.02% (consisting of 65.28% of explained variance for the first component, and 27.74% of explained variance for the second component), showing an *R*^2^ equal to 0.926. The profiles of the calculated pure spectra and pure concentrations are reported in Figures 7A,B. The molar extinction coefficients of the two species (hereinafter, component 1 = MTA1, component 2 = MTA2) were also estimated: $\epsilon_{\max}^{MTA1}\;=\;3487\text{ mo}{\text{l}}^{-1}\text{ L }{\text{cm}}^{-1}\;\left(\lambda_{\max}\;=\;580\text{ nm}\right);\;\epsilon_{\max}^{MTA2}\;=\;7576\text{ mo}{\text{l}}^{-1}\text{ L c}{\text{m}}^{-1}\;\left(\lambda_{\max}\;=\;513\text{ nm}\right)$. The molar extinction coefficients of the species MTA1 is in good agreement with the values usually proposed for the bis-catecholate complexes (Sever and Wilker, 2004).

### Chemical Model

The impossibility to define a real molar concentration of TA hinders defining thermodynamic parameters to quantitatively describe the chemical system because these parameters are based on the ratio of the molar concentration of the species in solution. Since this condition is an analogous with HS, the “single-site model” proposed by Hummel for HS (Hummel, 1997) was borrowed in this work to formalize the Fe(III)–TA interaction. Based on this model, a conditional formation constant that quantitatively expresses the strengthening of the interaction between the TA and the metal cation can be expressed as (Equation 4):

(4) $$\begin{array}{l} {\;}^{c}K=\frac{\left[MTA\right]}{\left[M\right]\;{\left(TA\right)}_{total}} \end{array}$$

where [*MTA*] is the molar concentration of the metal bound to TA, [*M*] is the molar concentration of the free metal cation, and (*TA*)_*total*_ is the concentration of total TA in solution expressed as mass per unit of volume (g L^−1^). The ^*c*^*K* is a conditional value that depends on both pH and solution component concentration. The simplest model proposed by Hummel is based on six assumptions (Hummel, 1997): (1) The metal ion M forms only 1:1 complexes with ligand sites L of the macro-molecule. The macro-molecule has several functional groups S. The number of S is not specified in the model but is assumed to be fixed within the pH and metal concentration range where the model is applied. (2) For each metal ion M under study, only one kind of ligand site L predominates within the parameter range where the model is used. (3) The complexing strength of the ligand sites L is constant and does not vary with the location within the macro-molecule; i.e., the influence of different substituents and varying stereochemistry on L is ignored. (4) Chemical changes of the macro-molecules have no influence on the number of active ligand sites available for metal complexation. (5) The functional groups involved in metal binding do not undergo any proton exchange reactions in the pH range of interest. (6) There are no interactions between functional groups S of the macro-molecule, i.e., electrostatic effects that change the binding characteristics of S are ignored.

Based on the model, the concentration of the macro-molecules is in relation with the concentration of the ligand sites through the Site Complexation Capacity (*SCC* – mol g^−1^),

(5) $$\begin{array}{l} {\left(TA\right)}_{total}\cdot SCC={\left[L\right]}_{total} \end{array}$$

The *SCC* can be defined also as the maximum number of moles of metal cation bound per unit of mass of macro-molecule at metal saturation. For the case under study, the *SCC* can be defined considering the results obtained by Job's plot method. Each mole of TA can bind four moles of Fe(III); therefore, the *SCC* results in 2.35 × 10^−3^ mol of Fe(III) per gram of TA (Figures 3A,C) and, for a nominal molar TA concentration of 1.0 × 10^−5^ mol L^−1^, the concentration of the ligand sites is [*L*]_*total*_ = 4.0 × 10^−5^ mol L^−1^. Based on the spectral features and on the value estimated for [*L*]_*total*_, it is reasonable interpret that the nature of L should correspond to a bis-catecholate group.

**Figure 8:**
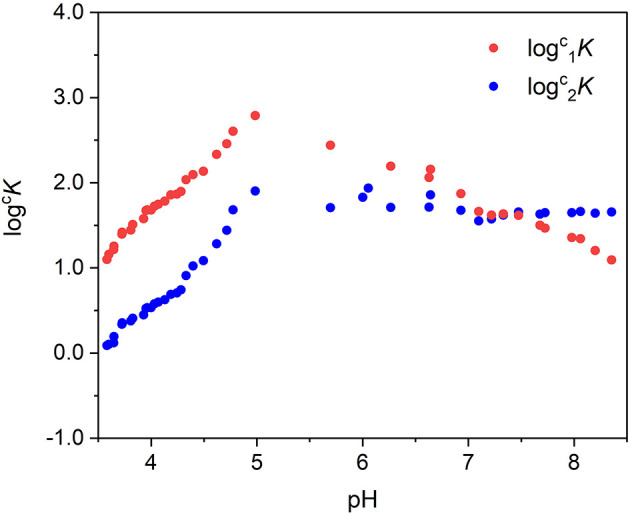
Values of $\log_{n}^{c}K$ as a function of pH (some experimental points corresponding to the curve inflection were excluded).

The concentration of the species in solution estimated by MCR-ALS, for each pH value, was hence used to calculate ^*c*^*K* applying Equation (4). Two conditional formation constants were defined, one for each of the two species detected, and Equations (6) and (7) were used:

(6) $$\begin{array}{l} {\;}_{1}^{c}K=\frac{\left[MTA1\right]}{\left[M\right]\;{\left(TA\right)}_{total}} \end{array}$$

(7) $$\begin{array}{l} {\;}_{2}^{c}K=\frac{\left[MTA2\right]}{\left[M\right]\;{\left(TA\;\right)}_{total}} \end{array}$$

where [MTA1] and [MTA2] are the concentrations of the two complex species, defined by MCR-ALS, and [M] is the concentration of the non-complexed metal cation. The values of log^*c*^*K* obtained were then plotted as a function of pH (Figure 8). The log^*c*^*K* linearly increases with pH until pH ~5. This behavior is expected because the assumption number 5 of the Hummel's model, which asserts that the functional groups L involved in metal binding do not undergo any proton exchange reactions in the pH range of interest, is not true for TA. In the pH range 3.5–5.0, the coordination of the binding sites involves the proton exchange reaction and at least a fraction of binding sites dissociates for competition of metal cation toward the proton. On the other hand, the pH 5 corresponds to the maximum formation of the first species and to the start point of formation of the second one, and between pH 4.5–6, it is possible to observe an inflection point of the alkalimetric titration.

Rearranging Equations (6) and (7) as reported below (Equation 8), and combining Equation (8) with the mass balance (Equation 9), it is possible to define the concentration of the complex species as a function of the total concentration of the cation ad of the total amount of TA:

(8) $$\begin{array}{l} {\;}_{n}^{c}K\;{\times \left(TA\right)}_{total}=\frac{\left[MTAn\right]}{\left[M\right]\;}{=}_{n}^{c^{\prime }}K\; \end{array}$$

(9) $$\begin{array}{l} {\left(M\right)}_{total}=\left[M\right]+\left[MTA1\right]+\left[MTA2\right] \end{array}$$

(10) $$\begin{array}{l} \left[MTAn\right]=\left(\frac{{\;}_{n}^{c^{\prime }}K}{{\;}_{1}^{c^{\prime }}K{+}_{2}^{c^{\prime }}K+1}\right){\left(M\right)}_{total} \end{array}$$

With the absorbance being proportional to the concentration of the chromophore and having additive property, it is possible to write Equation (10) as follows:

(11) $$\begin{array}{l} Abs^{\lambda }\;=\left(\varepsilon_{MTA1}^{\lambda }\left(\frac{{\;}_{1}^{c^{\prime }}K}{{\;}_{1}^{c^{\prime }}K{+}_{2}^{c^{\prime }}K+1}\right)+\varepsilon_{MTA2}^{\lambda }\left(\frac{{\;}_{2}^{c^{\prime }}K}{{\;}_{1}^{c^{\prime }}K{+}_{2}^{c^{\prime }}K+1}\right)\right){\left(M\right)}_{total} \end{array}$$

By the application of Equation (11), it was possible to foresee the absorbance values for solutions containing Fe(III) and TA having a metal-to-ligand ratio 1:1, at a defined pH. Equation (11) was employed to calculate the absorbance at pH 4.0 of the solutions containing increasing amount of metal cation to validate the chemical model proposed. The parameters used for the estimation of $Abs_{pH=4}^{570}$ were: $\varepsilon_{MTA1}^{570}=3,472\text{ mo}{\text{l}}^{-1}\text{L c}{\text{m}}^{-1}$, $\varepsilon_{MTA2}^{570}=6,419\text{ mo}{\text{l}}^{-1\;}\text{L c}{\text{m}}^{-1}$, ${\;}_{1}^{pH\;4}K=52.9\;L\;{\text{g}}^{-1}$, ${\;}_{2}^{pH\;4}K=3.78\;L\;{\text{g}}^{-1}$, (*TA*)_*total*_, and (*M*)_*total*_. The relative differences between experimental and calculated values ranged between 12 and 5.2%, revealing a quite good prediction capability.

## Discussion

The pH range in which the system Fe(III)–TA is not affected by redox reactions and the binding capability of TA toward Fe(III) were defined. The UV-vis spectrophotometry and the chemometric tools used to analyze the spectroscopic data recorded on the metal–ligand system turned out useful to interpret the chemistry of the system and allowed to evaluate the spectral features and the concentration profiles of the species in solution as a function of pH. In particular, the application of the MCR-ALS technique allowed obtaining quantitative information. The impossibility to define a molar concentration of the ligand prevents the use of hard-modeling methods for the analysis of the dataset; moreover, the necessity to work with a very restricted concentration scale limits the range and the techniques of investigation. These conditions strongly affect the possibility to define a speciation model and to achieve thermodynamic parameters that explain the chemistry of the system, but the approach here used enabled us to quantitatively interpret the behavior of Fe(III) in the presence of TA. The capacity of MCR-ALS to apply constraints contributed to define the chemical meaning of the mathematical solutions. Then, the MCR-ALS results were used to formalize the parameters that control the chemical system having the possibility to overcome the definition of an exact stoichiometry and a molar concentration of the ligand through the application of the chemical modeling proposed for HS.

The combination of the chemometric tools with the formalism used to interpret the metal cation interaction with HS could be a useful way to model those chemical systems in which the ligands are characterized by a not well-defined mass. This work reports a first exploratory application and the methodology needs to be tested further on different chemical systems, but the development of this approach could lead to formulate models with a good predictive capacity as it is already possible to do for the chemical systems that treat low-molecular-mass ligands.

## Data Availability Statement

The original contributions presented in the study are included in the article/supplementary material, further inquiries can be directed to the corresponding author/s.

## Author Contributions

SB devised and designed the study. EA dealt with the application of the chemometric techniques. SB and EA contributed to the data collection, interpretation, both participated in the writing, and editing of the manuscript.

## Conflict of Interest

The authors declare that the research was conducted in the absence of any commercial or financial relationships that could be construed as a potential conflict of interest.
